# Crystal structure of the conserved domain of the DC lysosomal associated membrane protein: implications for the lysosomal glycocalyx

**DOI:** 10.1186/1741-7007-10-62

**Published:** 2012-07-19

**Authors:** Sonja Wilke, Joern Krausze, Konrad Büssow

**Affiliations:** 1 Department of Molecular Structural Biology, Helmholtz Centre for Infection Research, Inhoffenstr. 7, 38124 Braunschweig, Germany

## Abstract

**Background:**

The family of lysosome-associated membrane proteins (LAMP) comprises the multifunctional, ubiquitous LAMP-1 and LAMP-2, and the cell type-specific proteins DC-LAMP (LAMP-3), BAD-LAMP (UNC-46, C20orf103) and macrosialin (CD68). LAMPs have been implicated in a multitude of cellular processes, including phagocytosis, autophagy, lipid transport and aging. LAMP-2 isoform A acts as a receptor in chaperone-mediated autophagy. LAMP-2 deficiency causes the fatal Danon disease. The abundant proteins LAMP-1 and LAMP-2 are major constituents of the glycoconjugate coat present on the inside of the lysosomal membrane, the 'lysosomal glycocalyx'. The LAMP family is characterized by a conserved domain of 150 to 200 amino acids with two disulfide bonds.

**Results:**

The crystal structure of the conserved domain of human DC-LAMP was solved. It is the first high-resolution structure of a heavily glycosylated lysosomal membrane protein. The structure represents a novel β-prism fold formed by two β-sheets bent by β-bulges and connected by a disulfide bond. Flexible loops and a hydrophobic pocket represent possible sites of molecular interaction. Computational models of the glycosylated luminal regions of LAMP-1 and LAMP-2 indicate that the proteins adopt a compact conformation in close proximity to the lysosomal membrane. The models correspond to the thickness of the lysosomal glycoprotein coat of only 5 to 12 nm, according to electron microscopy.

**Conclusion:**

The conserved luminal domain of lysosome-associated membrane proteins forms a previously unknown β-prism fold. Insights into the structure of the lysosomal glycoprotein coat were obtained by computational models of the LAMP-1 and LAMP-2 luminal regions.

## Background

Lysosomes of mammalian cells typically appear as small, spherical bodies with amorphous, electron-dense cores and a single limiting membrane. The interior of lysosomes is acidic at pH 4.8. Lysosomes contain an array of hydrolases with acidic pH optimum that can degrade all cellular macromolecules. Three mechanisms provide substrates for lysosomal degradation: endocytosis, autophagy and phagocytosis.

Newly synthesized lysosomal proteins exit the trans-Golgi network in vesicles which fuse with endosomes [1]. Soluble lysosomal enzymes are tagged with mannose-6-phosphate and are sorted in the Golgi compartment by mannose-6-phosphate receptors. Lysosomal membrane proteins have sorting signals in their cytosolic domains. Early endosomes acquire lysosomal proteins and an acidic pH during their maturation to late endosomes [2]. Lysosomes can be distinguished from endosomes by the absence of mannose-6-phosphate receptors, a lower internal pH and a distinct morphology [3]. Lysosomes appear electron dense by comparison with endosomes and can be separated by density gradient centrifugation. Lysosomes may be regarded as storage organelles for degradative enzymes, with the degradation of substrates occurring mainly in hybrid organelles of late endosomes and (auto)phagosomes [4]. Endosomes deliver endocytosed molecules from the extracellular space for degradation, and also proteins provided by the ESCRT pathway. ESCRT complexes package ubiquitinated proteins into vesicles formed by inward budding of the endosomal limiting membrane. Fusion of a late endosome with lysosomes creates a hybrid organelle of intermediate density [5]. The hybrid organelle hydrolyses its cargo - macromolecules and intraluminal vesicles - and transports the small molecular products into the cytosol. The remaining lysosomal components are condensed and form new dense-core lysosomes [6].

The lysosome's degradative machinery is potentially harmful to its limiting membrane, since it is capable of degrading intact lipid membranes. The inside of the lysosomal membrane is protected from degradation, presumably by a high abundance of heavily glycosylated membrane proteins. The membrane is quickly degraded when its glycoconjugate-free outside is exposed to the lysosomal interior [7].

Lysosomes are characterized by a high abundance of heavily glycosylated membrane proteins. These proteins form a dense coat on the inside of the lysosomal membrane. The glycoprotein coat can be visualized by electron microscopy as a thin, electron translucent halo separating the membrane from the condensed core [8,9]. It can be stained by glycoprotein-specific reagents and its thickness ranges from 5 to 12 nm with an average of 8 nm [8,9]. In comparison, the glycocalyx on the cell surface is much thicker, up to several micrometers [10]. Intracellular glycoprotein membrane coats were observed in lysosomes, endosomes, autophagosomes and secretory granules [9]. They are believed to protect vesicle membranes by limiting the access of degradative factors to the lipid bilayer.

**Figure 1:**
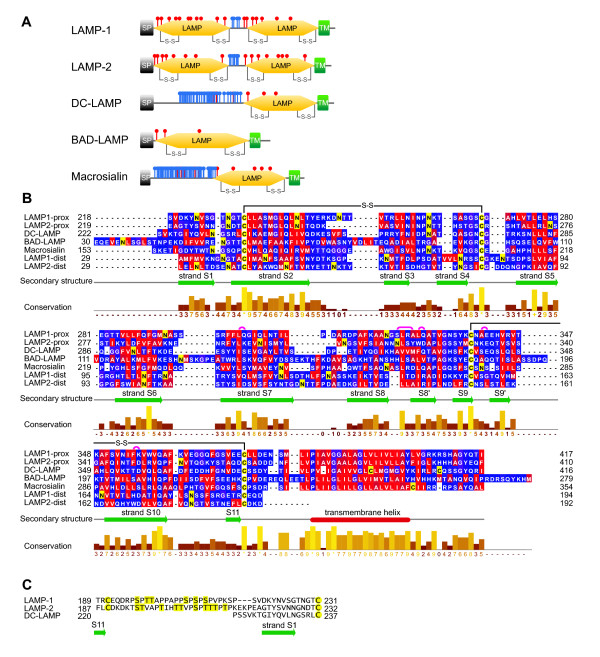
**The LAMP protein family**. (**A**) Domain architectures of the five human LAMP family proteins (UniProt P11279, P13473, Q9UQV4, Q9UJQ1, P34810). O- and N-linked glycosylation sites are indicated in blue and red, respectively. SP, signal peptide; TM, transmembrane helix. (**B, C**) Sequence alignments of the seven human LAMP domains and adjacent transmembrane domains (B) and of the 'hinge' region (linker) connecting the membrane-distal ('dist') and membrane-proximal ('prox') domains of LAMP-1 and LAMP-2 (C). Glycosylated residues are displayed with yellow background. Residues involved in β-bulges are indicated by arcs in magenta. Hydrophobic residues are colored red and hydrophilic ones are colored blue (in B). S-S: disulfide bond.

The major lysosomal membrane proteins, the homologous LAMP-1 (lgp120, CD107a) and LAMP-2 (lgp110, CD107b), are the standard markers for the lysosomal compartment [11]. They are among the most extensively glycosylated proteins with glycan chains that outweigh the protein core and that include high-molecular-weight poly-N-acetyllactosaminoglycans [12]. LAMP-1 and 2 are type-I membrane proteins of similar length and identical domain structure. The transmembrane region is followed by a short, C-terminal cytosolic tail that comprises motifs for lysosomal targeting [1]. The luminal region comprises two similar N-glycosylated domains of about 160 residues, each with two conserved disulfide bonds (Figure 1). The two domains are separated by a proline-rich, O-glycosylated 'hinge' region of about 30 amino acid residues [13].

LAMP-1 and LAMP-2 are abundant proteins, representing 0.1 to 0.2% of total cell protein [14]. They are enriched in lysosomes, late endosomes and mature (auto)phagosomes. Both proteins are ubiquitous in human tissues and cell types [15]. Their expression is particularly pronounced in metabolically active cells. The *LAMP-2 *gene has three splice forms, LAMP-2A, -2B, -2C, which differ in the transmembrane and cytosolic regions [15]. The *LAMP-1 *gene encodes for a single transcript.

Considerable knowledge on LAMP function was derived from *LAMP-1 *and *LAMP-2 *gene knockout mice [16,17] and *LAMP-1/2*-negative cell lines [18]. Impaired fusion of phagosomes and autophagosomes with lysosomes was observed. *LAMP-1 *and *2 *can complement each other to a large extent. Double *LAMP-1/2 *knockout leads to embryonic lethality. In contrast, *LAMP-1 *knockout mice are healthy [16]. They display upregulated levels of LAMP-2. Knockout of the *LAMP-2 *gene leads to elevated postnatal mortality [17]. Surviving mice have a phenotype that corresponds to Danon disease, a rare genetic condition caused by LAMP-2 deficiency [19]. Autophagosomes accumulate in several tissues of LAMP-2 negative mice. Danon disease entails muscle weakness, heart disease and mental retardation. It is associated with disturbed autophagosome maturation and extensive accumulation of autophagosomes in muscle cells.

LAMP proteins are important regulators of lysosome fusion with autophagosomes and phagosomes [20]. Macrophages lacking either LAMP-1 or LAMP-2 are capable of normal phagocytosis. However, knockout of both genes interferes with fusion of phagosomes with lysosomes [21,22]. Phagosomes lacking both LAMP-1 and 2 did not move from the periphery of the cell towards the perinuclear lysosomes [21]. In contrast to macrophages, LAMP-1 cannot compensate for lack of LAMP-2 in neutrophil phagocytes. *LAMP-2 *knockout mice suffer from a high prevalence of periodontitis because their neutrophils cannot keep the responsible bacteria in check [23].

LAMP-2 has been implicated in cholesterol transport from the lysosome [18,24]. It was shown that the membrane-proximal luminal domain of LAMP-2 is specifically required to rescue the cholesterol transport deficiency of *LAMP-1/2 *double knockout cells [24].

LAMP-1 and 2 are major components of the glycoconjugate coat on the inside of the lysosomal membrane. It was estimated that the concentration of LAMPs is sufficiently high for the formation of a nearly continuous layer on the inner surface of the lysosomal membrane [25]. It was, therefore, expected that their removal would destabilize lysosomes. However, depletion of LAMP-1 and 2 had no detectable effect on lysosome integrity. Removal of N-linked glycans with endoglycosidase H caused rapid degradation of LAMP-1 and LAMP-2, but did not destabilize lysosomes [26]. Lysosomes of *LAMP-1/2 *knockout cells have abnormalities, but their limiting membranes are apparently intact [18]. Other glycoproteins and glycolipids might have provided sufficient membrane protection in these studies. Lysosomal hybrid organelles with larger limiting membrane surface and lower membrane glycoprotein density might be more dependent on LAMPs for protecting their membranes.

Intracellular glycoconjugate coats are found on the membranes of secretory granules [9]. During degranulation of Natural Killer (NK) or cytotoxic T-cells, cytotoxic effectors become activated, which can potentially harm the effector cells' cell membrane. LAMP-1 is a surface marker of NK cells that have degranulated. It can protect NK cells against their own cytotoxic effectors following degranulation (André Cohnen, University of Heidelberg, Carsten Watzl, Leibniz Research Centre for Working Environment, unpublished data).

Chaperone-mediated autophagy (CMA) is a lysosomal pathway for selective removal of damaged cytosolic proteins (reviewed in [27]). CMA effects direct transmembrane import of cytosolic proteins into the endolysosomal system. The LAMP-2 isoform LAMP-2A functions as a receptor for cytosolic proteins and also as essential component of the CMA translocation complex [28]. Cytosolic substrate proteins bind to monomers of LAMP-2A, which then multimerizes to form the complex required for substrate transmembrane import. Membrane-associated molecules of hsc70 actively disassemble LAMP-2A into monomers to initiate a new cycle of binding and translocation [28]. Expression of LAMP-2A normally declines in the liver of mice as they age. Genetic engineering for overexpression of LAMP-2A in the liver not only restored CMA, but also (macro)autophagy and proteasomal pathways to the levels observed in young animals. As a result, the age-related decline of liver function was significantly reduced [29].

The five human members of the LAMP protein family are characterized by a conserved 'LAMP domain' directly adjacent to the single transmembrane helix (Figure 1). LAMP-1 and 2 are ubiquitous proteins, whereas DC-LAMP (LAMP-3) [30], BAD-LAMP (UNC-46, C20orf103) [31] and macrosialin (CD68) [32] are only expressed in specific cell types.

Dendritic cells (DCs) are professional antigen-presenting cells with the task of activating immune responses. DC-LAMP levels increase progressively during the differentiation of human DCs from hematopoietic bone marrow progenitor cells. DCs search for pathogens in tissues in contact with the external environment. They phagocytose pathogens, become activated and migrate to lymph nodes where they present pathogen-specific antigens on their cell surface using MHC class II molecules. DC-LAMP levels rises steeply upon activation of human DCs [30]. The protein co-localizes with MHC class II molecules in an intracellular compartment. DC-LAMP is a highly specific marker for mature DCs in humans and other mammals including cattle and pigs, but it is not expressed in DCs of mice [33].

Mammals, including mice, have a second cell type with high DC-LAMP expression: type II pneumocytes [34]. These cells secrete the lung's surfactant and, like DCs, can present antigens of pathogens on MHC class II molecules [35,36]. DC-LAMP co-localizes with MHC class II molecules on the limiting membrane of surfactant protein-containing organelles [34]. In chicken, DC-LAMP expression is upregulated upon DC activation [37]. In contrast to mammals, DC-LAMP was found in nearly all chicken tissues tested.

The structural basis of the various and seemingly unrelated functions of the lysosome associated membrane proteins is currently unclear. No information about the three-dimensional structures of any of the highly glycosylated proteins that form the inner lysosomal membrane coat were available before this study and the structural basis for the electron microscopical appearance of the coat has remained unclear. Previously, we established a novel expression system for glycosylated proteins. With this system, stable cell lines for a number of LAMP domains were established and crystals of the membrane-proximal domain of human DC-LAMP (LAMP-3) were obtained [38,39]. Here we report the structure solved with these crystals. Unexpected structural features and possible sites of molecular interactions were uncovered. *In silico *models of LAMP-1 and LAMP-2 were generated that allowed us to draw conclusions on the structure of the lysosomal membrane coat.

## Results

### The DC-LAMP domain has a novel β-prism fold

The crystal structure of the membrane-proximal domain of human DC-LAMP (Figure 1) was solved by multi-wavelength anomalous dispersion (MAD) phasing and refined at a resolution of 2.8 Å (Table 1). Two protein molecules denoted chain A and B are present in the asymmetric unit, which are related to each other by a two-fold non-crystallographic symmetry. Analysis with the *protein interfaces, surfaces and assemblies service PISA *[40] predicted that the domain is a monomer in solution, in correspondence with gel filtration results. Chains A and B have almost identical conformations except for one loop, which adopts two distinct conformations (discussed below). This is reflected by an r.m.s. distance for the aligned chains' C_α _atoms of 0.39 Å if this loop is excluded and of 2.1 Å if the loop is included. If not otherwise noted, the following sections refer to chain A, which is based on a better defined electron density map.

**Table 1:** Data collection and refinement statistics.

Data collection			
Beamline	DESY X12		

Temperature (K)	100		

Dataset type	Peak	Inflection point	High-energy remote

Wavelength (Å)	1.10371	1.10420	1.10009

Data range (°)	180		

Oscillation range (°)	0.5		

Space group	P3_1_		

Unit cell parameters (Å)	a = b = 53.0, c = 143.5		

Resolution (Å)	20 to 2.8 (2.95 to 2.8)		

Unique reflections	19,800 (3,352)	19,790 (3,392)	19,765 (3,365)

Redundancy	2.84 (2.84)	2.83 (2.82)	2.84 (2.84)

Completeness (%)	90.0 (94.5)	89.9 (96.9)	89.7 (95.9)

Mean I/σI	16.5 (3.45)	15.52 (2.92)	18.19 (3.84)

*R*_merge_^a^	0.05 (0.30)	0.06 (0.40)	0.05 (0.28)

Mosaicity (°)	0.271		

Estimated Wilson *B *(Å^2^)	54.1		

**Refinement**			

*R_work_*^b^	0.2271		

*R_free_*^c^	0.2508		

Molecules in the asymmetric unit (DC-LAMP/GlcNAc/Ir)	2/2/4		

No. of atoms			

Protein	2,438		

Hetero atoms	32		

Water	10		

Total	2,480		

Atomic displacement factor *B *(Å^2^)	61.3		

Real space correlation coefficient^d^	0.882		

r.m.s.d. from ideal			

Bond lengths (Å)	0.0056		

Bond angles (°)	1.023		

Ramachandran plot			

Favored (%)	90.9		

Allowed (%)	6.8		

Disallowed (%)	2.3		

Values in parentheses account for the highest resolution shell.

^a ^$R_{merge}=\sum_{hkl}\sum_{i}\left|I_{hkl,i}-\bar{I_{hkl}}\right|/)\sum_{hkl}\sum_{i}I_{hkl,i}$

^b ^$R_{work}=\sum_{hkl}\left|\left|F_{obs}\right|-\left|F_{calc}\right|\right|/)\sum_{hkl}\left|F_{obs}\right|$

^c ^*R_free _*is calculated as *R_work _*but using *F_obs _*derived from 5% randomly selected reflections exclude from refinement.

^d ^$RSCC=\underset{xyz}{\sum }\left|\rho_{o}-\bar{\rho_{o}}\right|\underset{xyz}{\sum }\left|\rho_{c}-\bar{\rho_{c}}\right|/{\left({\underset{xyz}{\sum }\left|\rho_{o}-\bar{\rho_{o}}\right|}^{2}{\underset{xyz}{\sum }\left|\rho_{c}-\bar{\rho_{c}}\right|}^{2}\right)}^{1/2}$

**Figure 2:**
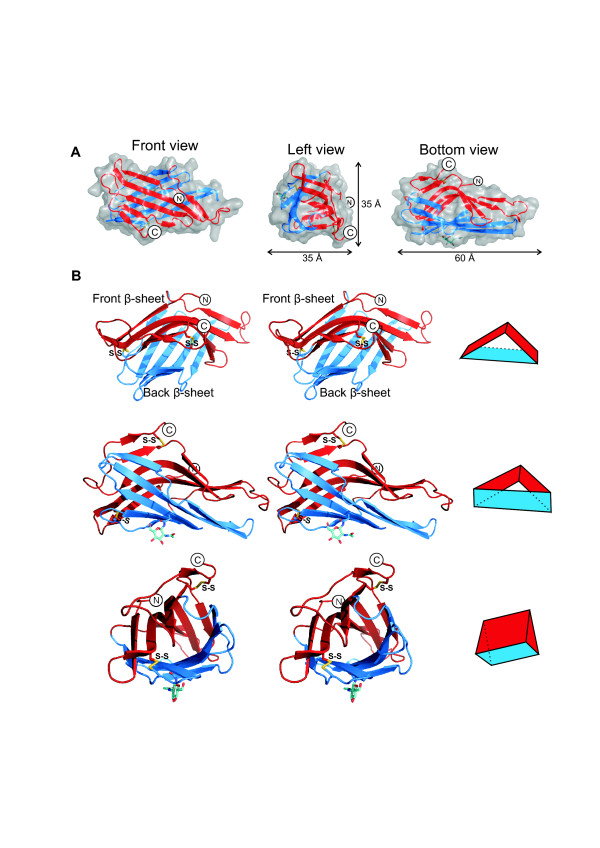
**The structure of the DC-LAMP domain**. (**A**) Different views of the domain's shape. The two β-sheets are drawn in red and blue. (**B**) Stereo views of the domain's β-prism shape. Schematic prism shapes are drawn on the right for orientation. The N-acetyl-glucosamine residue is depicted as a stick drawing in cyan.

**Figure 3:**
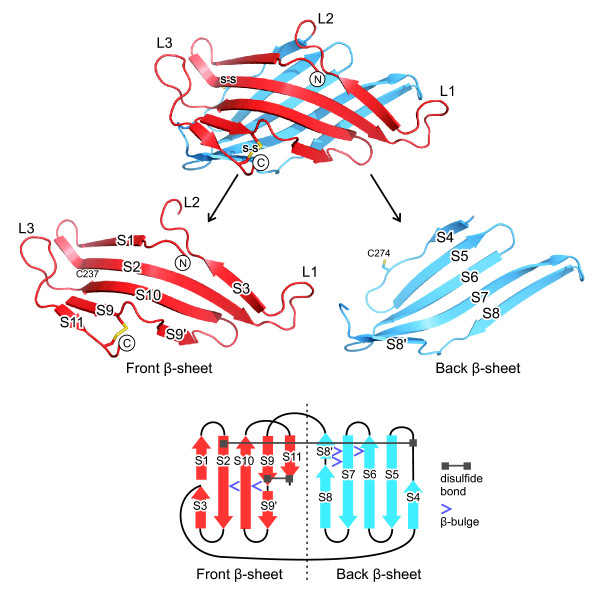
**The β-strand arrangement of the DC-LAMP domain**. The 'front' and 'back' β-sheets are drawn in red and blue, respectively. The cysteines that form the first disulfide bond (S-S) are labelled. β-strands and loops are identified as S1 to S11 and L1 to L3, respectively. The topology of the β-sheets is drawn schematically in the lower part with sheets opened out.

The DC-LAMP domain consists of two β-sheets that form a "pseudo β-prism" (Figure 2). According to the SCOP database [41], the pseudo β-prism fold consists of a β-sandwich with one regular β-sheet and the other β-sheet bent in the middle with a set of aligned β-bulges. The triangular base of the domain's prism shape is 60 Å wide and 35 Å high and the prism's height is 35 Å. The domain's N- and C-termini are located on the 'front' β-sheet, which consists of 6 β-strands (Figure 3). β-strands S1 and S3 are short and arranged in tandem, antiparallel to strand S2. Two more antiparallel β-strands, S10 and S9, are followed by the short C-terminal β-strand S11, which is arranged in parallel to S9.

**Figure 4:**
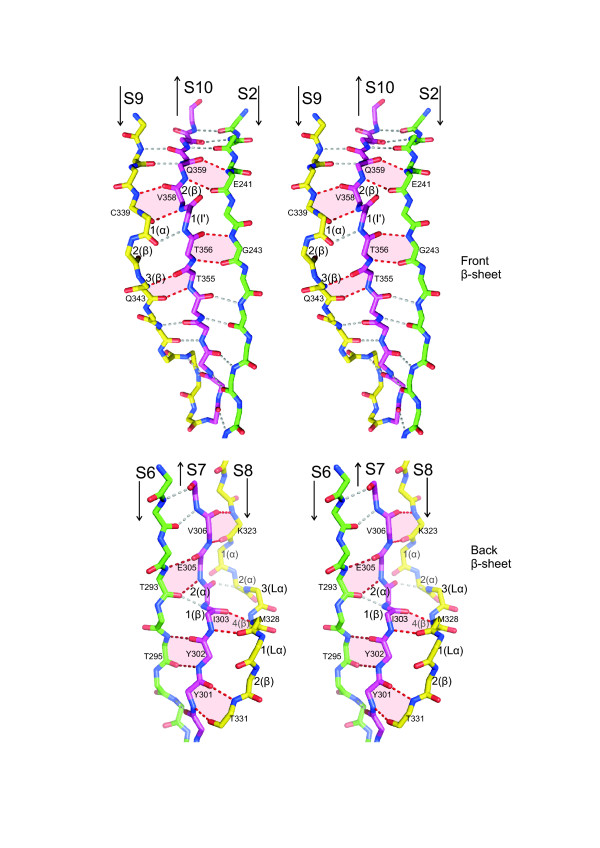
**Stereo views of the β-bulges**. The β-bulges that bend the front and back β-sheets are shown. Main chain atoms of parts of β-strands are shown as stick drawings. Bulged residues are numbered and their conformations are given in parentheses (α, α-helix; β, β-strand; I', type I' β-turn; Lα, left-handed α-helix). Hydrogen bond pairs that flank β-bulges are shown in red and the corresponding residues are labelled. Other inter-main chain hydrogen bonds are drawn in grey.

**Figure 5:**
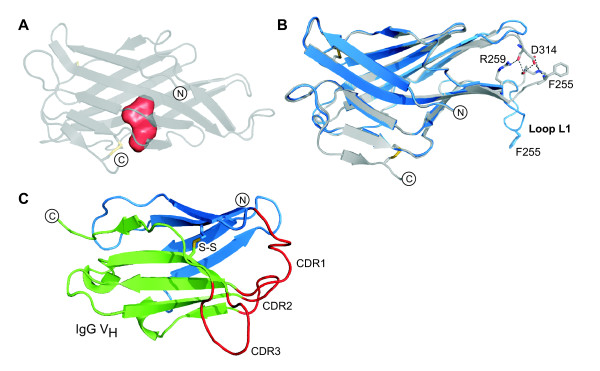
**The hydrophobic pocket and the variable loop conformation**. (**A**) The hydrophobic pocket in the DC-LAMP domain was filled with water molecules, which are displayed as a red surface, using HOLLOW [70]. (**B**) Structural alignment of DC-LAMP chain A (grey) and B (blue). Hydrogen bonds connect loop L1 to an adjacent hairpin in chain A, but not in chain B. (**C**) A V_H _immunoglobulin domain is shown for comparison (PDB 1IGT). Complementarity determining regions (CDR) are drawn as red loops. The two β-sheets of the domain, displayed in green and blue, are connected by a disulfide bond (S-S).

The front β-sheet is bent in the middle by two connected β-bulges [42], which are formed by the β-strand pairs S2/S10 and S10/S9 (Figure 4). This bending forms the domain's triangular prism shape and it opens a hydrophobic pocket between the sheets, constituting a possible binding site for small molecules (Figure 5A).

The 'back' β-sheet consists of five antiparallel β-strands (Figure 3). Strand S4 comprises only four residues, 268 to 271, but the polypeptide chain continues in a β-strand-like conformation up to residue 275. It is distorted by the disulfide bond formed by cysteines 274 and 311. The back β-sheet is flat, except for the lower left corner, which is bent as if to close the opening of the domain created by the bending of the front β-sheet (Figure 2B). The bending of the back β-sheet is caused by three β-bulges between strands S6, S7 and S8 (Figure 4).

The domain's β-strands are connected by β-turns and three loops. Loop L2 links the front β-sheet with the back β-sheet (Figure 3). Two more loops of 6 and 10 residues connect strands S2 and S3 (Loop L1) and S10 and S11 (Loop L3), respectively. The conformation of loop L1 and the adjacent β-hairpin formed by strands S7 and S8 differs between chains A and B, due to crystal contacts, indicating that these regions are flexible in solution (Figure 5B). Several hydrogen bonds link loop L1 and the hairpin in chain A, but not in chain B.

According to the SCOP database [41], only two proteins are known to contain pseudo β-prism domains, a carbohydrate receptor binding protein of *Lactococcus lactis *phage P2 (UniProt Q71AW2, PDB 1ZRU, 2-140) [43] and a tail protein of *Bordetella *phage BPP-1 (UniProt Q775D6, PDB 1YU0, 5-170) [44]. The topology of the β-sheets (that is, the order of the β-strands) of the DC-LAMP domain differs from the other pseudo β-prism structures. The DC-LAMP domain therefore represents a novel fold (Alexey Murzin, personal communication).

Two disulfide bonds and an N-acetylglucosamine residue connected to Asn291 were visible in the DC-LAMP domain's electron density map (Figure 2B). The first disulfide bond stabilizes the β-prism by linking its two sheets. The second disulfide bond, linking the front β-sheet and the domain's C-terminus, strengthens the connection of the C-terminal transmembrane helix to the center of the front β-sheet. Interestingly, the N-acetylglucosamine at Asn291 participated in formation of the crystal lattice by forming a hydrogen bond to Gly287 of a neighboring protein molecule.

The LAMP domain shares features with immunoglobulin (Ig) domains (Figure 5C). Both domain types have an all-β fold of comparable size stabilized by a conserved disulfide bond between their two β-sheets. The variable Ig domains of antibodies contain three flexible loops forming the antigen-binding site, the complementarity determining regions (CDRs). Loop L1 and the adjacent β-hairpin of the LAMP domain are also flexible (Figure 5B) and might provide a site for specific molecular interactions.

### Models of glycosylated LAMP domains

**Figure 6:**
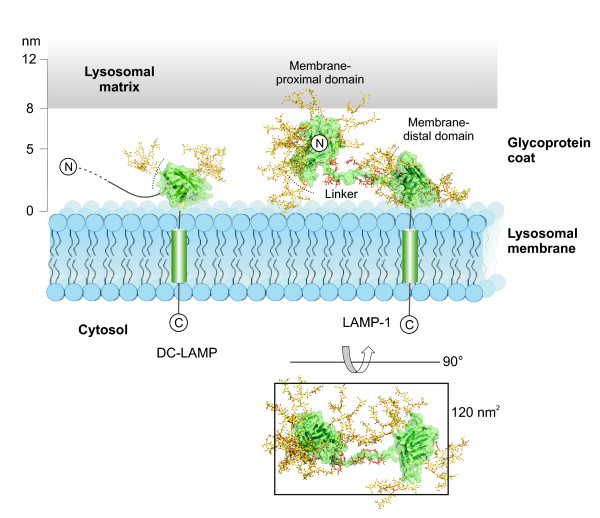
**Model of lysosomal membrane proteins and the glycoprotein coat**. Structural models of glycosylated DC-LAMP and LAMP-1 were drawn to scale. The models are based on the DC-LAMP crystal structure and a hypothetical *in silico *model of LAMP-1. The thickness of the glycoprotein coat was reported to range from 5 to 12 nm with an average of 8 nm. The membrane-distal, N-terminal domain of LAMP-1 may adopt other positions. Dotted lines indicate putative binding sites consisting of the flexible loop L1 and an adjacent β-hairpin (Figure 5B). A box corresponding to 120 nm^2 ^surface area was drawn around a top view of LAMP-1. Polypeptides are depicted in green and glycans in yellow.

The N-terminal segment of the luminal region of DC-LAMP is proline-rich and densely O-glycosylated (residues 28 to 221, Figure 1A), which implies an elongated, stiffened conformation due to steric restraints imposed by the glycan moieties [45]. A model of glycosylated full length DC-LAMP was drawn to scale with the domain's C-terminus pointing down towards the membrane (Figure 6). In this orientation, the protein's N-terminal segment is linked to the domain's side at about half height, suggesting an orientation of the N-terminal segment along the membrane.

The highly abundant LAMP proteins 1 and 2 are the main components of the thin coat present on the inside of lysosomal membranes. The conformation of their luminal domains in the coat is currently unclear. Structural models of the membrane-distal and membrane-proximal domains of LAMP-1 and LAMP-2 were generated by comparative modelling with [46] using DC-LAMP chain A as the template. Glycans of complex type were attached to the models with [47] and GLYCAM [48]. The resulting glycosylated domain structures had diameters of 6 to 9 nm. This corresponds to the thickness of the glycoconjugate coat of the lysosomal membrane, which was reported to range from 5 to 12 nm [8] (Figure 6).

**Figure 7:**
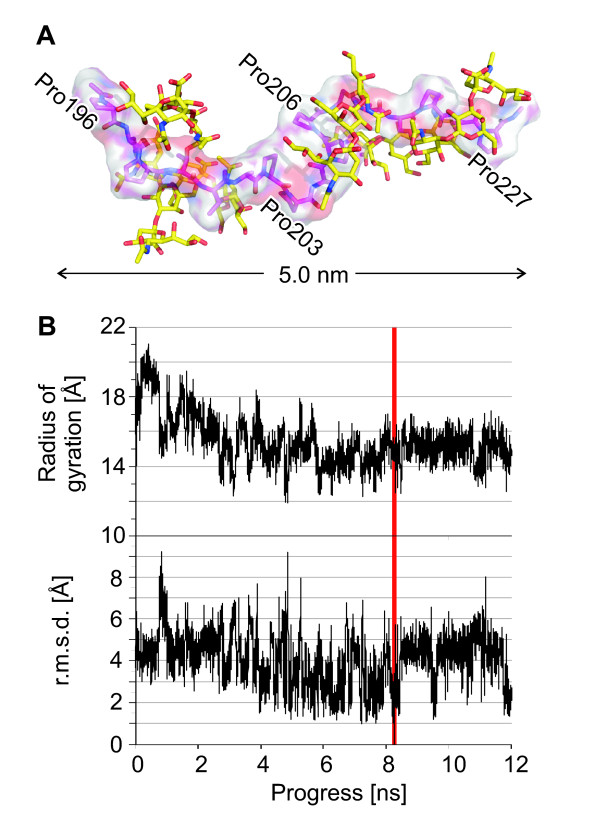
**Molecular modeling**. (**A**) The structure of the lowest energy obtained by molecular dynamics simulation is shown as a stick model and a van der Waals surface with pink carbon atoms. Glycans are shown with yellow carbon atoms. Prolines 203 and 206 have α-helical conformations. (**B**) Radius of gyration values during the simulation and r.m.s. deviations of the backbone atoms from the structure of lowest energy (marked in red). The peptide's lowest-energy structure had a radius of gyration of 14.6 Å, compared to 17.9 Å for the initial structure.

The two domains of LAMP-1 are connected by a linker of 23 residues, which comprises 11 prolines and 6 O-glycosylation sites in LAMP-1 (Figure 1C). This implies an elongated conformation lacking secondary structure and limited flexibility of the peptide backbone. The linker of LAMP-2 has similar length but more glycosylation sites. Conformational properties of the LAMP-1 linker were analysed by computational folding simulation. The sequence ^196^PSPTTAPPAPPSPSPSP^212 ^was subjected to a 12 ns simulation with AMBER force fields. The initial structure had an elongated β-strand conformation and carried sialylated O-glycans on the six Ser/Thr residues. During the simulation, folding was not observed (Figure 7). Residues departed from the initial β-strand conformation only transiently, except for prolines 203 and 206, which adopted α-helical conformations permanently. High values for the radius of gyration indicated elongated conformations throughout the simulation (Figure 7B). During the simulation, root-mean-square deviations of the backbone atoms from the lowest energy structure were in the order of 3 to 5 Å, indicated a high degree of structural flexibility (Figure 7B).

According to the *in silico *models, the N-termini of the membrane-proximal domains of LAMP-1 and LAMP-2 are localized on the side, as described above for the DC-LAMP domain, allowing their N-terminal, membrane-distal domains to localize close to the membrane. The models of the LAMP-1 domains and linker were combined into a model of the complete molecule (Figure 6). In the model, the dimensions of the luminal domain of LAMP-1 agree well with the reported thickness of the lysosomal glycoprotein coat. The model represents a possible conformation in which the linker is oriented horizontally and the N-terminal domain is located relatively close to the membrane. Alternative conformations with the linker pointing upwards appear less favorable due to steric hindrance and electrostatic repulsion of the sialic acid groups on the N- and O-linked glycans.

## Discussion

The crystal structure of the membrane-proximal domain of DC-LAMP reveals a novel β-prism fold. It is the first structure reported for the heavily glycosylated lysosomal membrane proteins. The structure proves that the proteinaceous core of these proteins can adopt a globular compact fold that is not fundamentally different from proteins with less glycosylation.

The structure explains the complete conservation of the pair of disulfide bonds in the LAMP protein family. The first disulfide bond stabilizes the β-prism, while the second disulfide bond fixes the domain's C-terminus to the center of the kinked β-sheet. The center of the sheet is thereby linked to the transmembrane helix. Loop L3, the flexible loop L1 and the adjacent β-hairpin point sideways. In this arrangement, the loops would be in a suitable position for interacting with luminal domains of neighboring membrane proteins.

The LAMP family is characterized by a conserved, membrane-proximal domain. Sequence conservation is not restricted to this domain, but includes the adjacent transmembrane helices (Figure 1B). All human LAMPs contain a conserved proline and two conserved glycine residues in the transmembrane helix. Glycine residues mediate interactions of transmembrane helices in membrane proteins [49]. In the course of chaperone-mediated autophagy (CMA), LAMP-2A oligomerizes into multimeric complexes. Mutation of the conserved glycines in the LAMP-2A transmembrane region to alanines inhibits oligomerization and significantly reduces CMA activity [28]. The lysosomal polypeptide transporter TAPL (ABCB9) has recently been identified as a specific binding partner of LAMP-1 and LAMP-2 [50]. The interaction appears to be mediated by transmembrane regions: the binding site for the LAMPs is localized in the N-terminal domain of TAPL, which comprises four transmembrane helices but no globular domains. The LAMP-2B isoform is recognized exclusively by TAPL, which differs from LAMP-2A only in the transmembrane region and the cytosolic tail. The structure solved in this study identified a possible protein binding site (Figure 5B). It is possible that LAMPs self-oligomerize or recognize other lysosomal membrane proteins by simultaneous interactions with their luminal and transmembrane regions. The LAMP family member unc-46 (BAD-LAMP) and the vesicular GABA transporter depend on each other for their vesicular sorting [51]. This phenomenon might be due to molecular interaction of the two proteins.

The dimensions of the *in silico *models are consistent with literature data on the thickness of the glycoprotein coat on the lysosomal membrane (Figure 6) and the density of LAMP molecules on the lysosomal membrane. In BHK cells, the surface area of lysosomal and LAMP-1 positive prelysosomal membranes was determined as 370 μm^2^/cell [52]. The average volume of the cells was 1,400 μm^3 ^[53]. LAMP-1 represents approximately 0.1% of the total cellular protein content of 200 to 250 mg/ml [25]. This amounts to a density of LAMP-1 in lysosomal membranes of approximately 2,600 molecules/μm^2^. The footprint of the LAMP-1 model in Figure 6 is about 120 nm^2^, which would implicate that about one third of lysosomal membranes is covered by LAMP-1. The model thus supports the concept of a continuous glycoprotein layer formed by LAMP-1, the similar LAMP-2 and other lysosomal proteins.

The luminal regions of lysosomal membrane proteins are typically small and do not contain more than a single folded domain. A comprehensive list of 45 lysosomal membrane proteins [54] was analysed with PFAM. Multidomain proteins were not found in this list and immunoglobulin or fibronectin domains, which are frequent in extracellular multidomain regions, were not present. This result corresponds to the thickness of the lysosomal glycoprotein coat of 8 nm on average, which is low in comparison to the glycocalyces on cell membranes of several hundred nanometers thickness [55].

It is generally believed that intracellular glycoprotein coats protect the limiting membranes of degradative vesicles against acid hydrolases. LAMP-1 protects natural killer cells against their own cytotoxic effectors. Besides protection, glycoconjugate coats on membranes of lysosomes and granules might be important in preventing attachment of the condensed content with the membrane, thereby supporting release of the content during phago-lysosomal fusion or exocytosis, respectively.

## Conclusion

This paper shows that the conserved domain of lysosome-associated membrane proteins forms a previously unknown β-prism fold with several unique structural features. The low thickness of the lysosomal inner membrane coat correlates with the compactness and membrane-proximity of the luminal regions of the heavily glycosylated lysosomal membrane proteins. Knowledge of the LAMPs' globular domains and their sites of possible molecular interaction will support continued research on the role of these proteins in the cell.

## Methods

### Multiple alignment, modelling

Multiple alignments were made with Jalview [56] and the L-INS-i method of MAFFT [57]. Comparative modelling of LAMP domains was performed with MODELLER (University of California, San Francisco, CA, USA) [46]. Complex type N-linked glycans were attached to structural models with the GLYPROT server [47]. The attached glycan was a 17-mer with 4 sialylated N-acetyllactosamine antenna (LinucsID 26155 in the Glycosciences glycan database [47]).

A structural representation of the linker ^196^PSPTTAPPAPPSPSPSP^212 ^between the domains of human LAMP-1 was created in an elongated conformation (Φ, Ψ approximately -82°, 125°) with PyMOL (Schrödinger LLC, New York, NY, USA) and Swiss-PdbViewer [58]. The sialylated trisaccharide αNeu5Ac(2-3)βGal(1-3)αGalNAc was attached to the 6 reported O-glycosylation sites [59] using GLYCAM [48]. Molecular dynamics simulations were performed with AMBER11 [60] and the GLYCAM06 force field [61] in an implicit generalized Born solvent. The initial structure was minimized by 500 steps of steepest descent followed by 500 of conjugate gradient. Then, the system was heated, with a time step of 0.5 fs, for 1 ps at 10 K, followed by a gradient of 4 ps to 300 K and an equilibration step of 500 ps at 300 K. The heated system was subjected to 12 ns simulation at 300 K with a time step of 2 fs and a heat bath coupling time constant of 0.5 ps.

### Protein production, crystallization, and heavy metal derivatization

The membrane-proximal domain of human DC-LAMP (UniProt Q9UQV4, 222-381) with a C-terminal His_6_-tag was produced with a stable CHO cell line, purified by nickel chromatography and gelfiltration, deglycosylated and crystallized as described [38,39]. Crystals were soaked for 0.5 to 10 minutes in 14 mM Ammonium hexachloriridate(III)hydrate (Hampton Research, Aliso Viejo, CA, USA) in mother liquor and immediately shock cooled in liquid nitrogen.

### Data collection and structure determination

Three sets of X-ray diffraction data were collected at beamline X12 of the EMBL outstation (Hamburg, Germany) at wavelengths of 1.10371, 1.10420 and 1.10009 Å which correspond to the peak, inflection point and high-energy remote of the Ir absorption spectrum, respectively. Data were processed with XDS [62] in space group P3_1 _to a high-resolution limit of 2.8 Å. The calculation of the Matthews coefficient [63] and a MOLREP self-rotation function [64] revealed the presence of two DC-LAMP molecules in the asymmetric unit. The phase problem was solved by multi-wavelength anomalous dispersion with Phenix.autosol of the Phenix software suite [65]. Phenix.autobuild was used to build an initial model of DC-LAMP but succeeded only in building parts of one of the two molecules in the asymmetric unit. This partial model was used as a search model for molecular replacement with Phaser [66], which resulted in the positions of both DC-LAMP molecules. The structure was completed by iterative steps of manual rebuilding in Coot [67] and refinement with Phenix.refine [65]. The complete data collection and refinement statistics are shown in Table 1. Superpositions were carried out and r.m.s. deviations were calculated with the UCSF Chimera package (University of California, San Francisco, CA, USA) [68]. The secondary structure was analyzed using the DSSP algorithm [69] and figures were prepared with PyMOL.

### PDB Accession Code

Coordinates and experimental structure factors have been deposited in the Protein Data Bank with accession code [PDB:4AKM].

## Abbreviations

BHK: baby hamster kidney; CDR: complementarity determining region; CHO: Chinese hamster ovary; CMA: chaperone-mediated autophagy; DC: dendritic cell; dist: membrane-distal; ESCRT: endosomal sorting complex required for transport; Gal: galactose; GalNAc: N-acetylgalactosamine; GlcNAc: N-acetylglucosamine; Ig: immunoglobulin; LAMP: lysosome-associated membrane protein; lgp: lysosomal membrane glycoprotein; Neu5Ac: N-acetylneuraminic acid; NK: natural killer; prox: membrane-proximal; r.m.s.(d.): root-mean-square (difference); RSCC: real space correlation coefficient; TAPL: transporter associated with antigen processing-like.

## Competing interests

The authors declare that they have no competing interests.

## Authors' contributions

SW prepared the derivatized crystals. JK and SW collected X-ray diffraction data sets. JK solved and refined the crystal structure. KB conceived of and coordinated the study, carried out the modeling and drafted the manuscript. All authors contributed to the final manuscript.

## References

[B1] Braulke T Bonifacino JS Sorting of lysosomal proteins Biochim Biophys Acta 2009 1793 605 614 10.1016/j.bbamcr.2008.10.016 19046998

[B2] Huotari J Helenius A Endosome maturation Embo J 2011 30 3481 3500 10.1038/emboj.2011.286 21878991 PMC3181477

[B3] Pillay CS Elliott E Dennison C Endolysosomal proteolysis and its regulation Biochem J 2002 363 417 429 10.1042/0264-6021:3630417 11964142 PMC1222494

[B4] Luzio JP Pryor PR Bright NA Lysosomes: fusion and function Nat Rev Mol Cell Biol 2007 8 622 632 10.1038/nrm2217 17637737

[B5] Bright NA Gratian MJ Luzio JP Endocytic delivery to lysosomes mediated by concurrent fusion and kissing events in living cells Curr Biol 2005 15 360 365 10.1016/j.cub.2005.01.049 15723798

[B6] Pryor PR Mullock BM Bright NA Gray SR Luzio JP The role of intraorganellar Ca^2+ ^in late endosome-lysosome heterotypic fusion and in the reformation of lysosomes from hybrid organelles J Cell Biol 2000 149 1053 1062 10.1083/jcb.149.5.1053 10831609 PMC2174832

[B7] Henell F Ericsson JL Glaumann H Degradation of phagocytosed lysosomes by Kupffer cell lysosomes Lab Invest 1983 48 556 564 6843086

[B8] Neiss WF A coat of glycoconjugates on the inner surface of the lysosomal membrane in the rat kidney Histochemistry 1984 80 603 608 6469717

[B9] Neiss WF Ultrachemistry of Intracellular Membrane Glycoconjugates 1986 Berlin, Heidelberg: Springer-Verlag 10.1007/978-3-642-71347-7 3535424

[B10] Ebong EE Macaluso FP Spray DC Tarbell JM Imaging the endothelial glycocalyx *in vitro *by rapid freezing/freeze substitution transmission electron microscopy Arterioscler Thromb Vasc Biol 2011 31 1908 1915 10.1161/ATVBAHA.111.225268 21474821 PMC3141106

[B11] Saftig P Saftig P Lysosomal membrane proteins Lysosomes 2005 New York: Springer 37 49

[B12] Carlsson SR Fukuda M The polylactosaminoglycans of human lysosomal membrane glycoproteins lamp-1 and lamp-2. Localization on the peptide backbones J Biol Chem 1990 265 20488 20495 2243102

[B13] Carlsson SR Fukuda M Structure of human lysosomal membrane glycoprotein 1. Assignment of disulfide bonds and visualization of its domain arrangement J Biol Chem 1989 264 20526 20531 2584229

[B14] Chen JW Pan W D'Souza MP August JT Lysosome-associated membrane proteins: Characterization of LAMP-1 of macrophage P388 and mouse embryo 3T3 cultured cells Arch Biochem Biophys 1985 239 574 586 10.1016/0003-9861(85)90727-1 3923938

[B15] Furuta K Yang X-L Chen J-S Hamilton SR August JT Differential expression of the lysosome-associated membrane proteins in normal human tissues Arch Biochem Biophys 1999 365 75 82 10.1006/abbi.1999.1147 10222041

[B16] Andrejewski N Punnonen EL Guhde G Tanaka Y Lullmann-Rauch R Hartmann D von Figura K Saftig P Normal lysosomal morphology and function in LAMP-1-deficient mice J Biol Chem 1999 274 12692 12701 10.1074/jbc.274.18.12692 10212251

[B17] Tanaka Y Guhde G Suter A Eskelinen EL Hartmann D Lullmann-Rauch R Janssen PM Blanz J von Figura K Saftig P Accumulation of autophagic vacuoles and cardiomyopathy in LAMP-2-deficient mice Nature 2000 406 902 906 10.1038/35022595 10972293

[B18] Eskelinen EL Schmidt CK Neu S Willenborg M Fuertes G Salvador N Tanaka Y Lullmann-Rauch R Hartmann D Heeren J von Figura K Knecht E Saftig P Disturbed cholesterol traffic but normal proteolytic function in LAMP-1/LAMP-2 double-deficient fibroblasts Mol Biol Cell 2004 15 3132 3145 10.1091/mbc.E04-02-0103 15121881 PMC452571

[B19] Nishino I Fu J Tanji K Yamada T Shimojo S Koori T Mora M Riggs JE Oh SJ Koga Y Sue CM Yamamoto A Murakami N Shanske S Byrne E Bonilla E Nonaka I DiMauro S Hirano M Primary LAMP-2 deficiency causes X-linked vacuolar cardiomyopathy and myopathy (Danon disease) Nature 2000 406 906 910 10.1038/35022604 10972294

[B20] Saftig P Beertsen W Eskelinen EL LAMP-2: a control step for phagosome and autophagosome maturation Autophagy 2008 4 510 512 18376150 10.4161/auto.5724

[B21] Huynh KK Eskelinen EL Scott CC Malevanets A Saftig P Grinstein S LAMP proteins are required for fusion of lysosomes with phagosomes Embo J 2007 26 313 324 10.1038/sj.emboj.7601511 17245426 PMC1783450

[B22] Binker MG Cosen-Binker LI Terebiznik MR Mallo GV McCaw SE Eskelinen EL Willenborg M Brumell JH Saftig P Grinstein S Gray-Owen SD Arrested maturation of Neisseria-containing phagosomes in the absence of the lysosome-associated membrane proteins, LAMP-1 and LAMP-2 Cell Microbiol 2007 9 2153 2166 10.1111/j.1462-5822.2007.00946.x 17506821

[B23] Beertsen W Willenborg M Everts V Zirogianni A Podschun R Schroder B Eskelinen EL Saftig P Impaired phagosomal maturation in neutrophils leads to periodontitis in lysosomal-associated membrane protein-2 knockout mice J Immunol 2008 180 475 482 18097049 10.4049/jimmunol.180.1.475

[B24] Schneede A Schmidt CK Holtta-Vuori M Heeren J Willenborg M Blanz J Domanskyy M Breiden B Brodesser S Landgrebe J Sandhoff K Ikonen E Saftig P Eskelinen EL Role for LAMP-2 in endosomal cholesterol transport J Cell Mol Med 2011 15 280 295 10.1111/j.1582-4934.2009.00973.x 19929948 PMC3822795

[B25] Granger BL Green SA Gabel CA Howe CL Mellman I Helenius A Characterization and cloning of lgp110, a lysosomal membrane glycoprotein from mouse and rat cells J Biol Chem 1990 265 12036 12043 2142158

[B26] Kundra R Kornfeld S Asparagine-linked oligosaccharides protect Lamp-1 and Lamp-2 from intracellular proteolysis J Biol Chem 1999 274 31039 31046 10.1074/jbc.274.43.31039 10521503

[B27] Kon M Cuervo AM Chaperone-mediated autophagy in health and disease Febs lett 2010 584 1399 1404 10.1016/j.febslet.2009.12.025 20026330 PMC2843772

[B28] Bandyopadhyay U Kaushik S Varticovski L Cuervo AM The chaperone-mediated autophagy receptor organizes in dynamic protein complexes at the lysosomal membrane Mol Cell Biol 2008 28 5747 5763 10.1128/MCB.02070-07 18644871 PMC2546938

[B29] Zhang C Cuervo AM Restoration of chaperone-mediated autophagy in aging liver improves cellular maintenance and hepatic function Nat Med 2008 14 959 965 10.1038/nm.1851 18690243 PMC2722716

[B30] de Saint-Vis B Vincent J Vandenabeele S Vanbervliet B Pin JJ Ait-Yahia S Patel S Mattei MG Banchereau J Zurawski S Davoust J Caux C Lebecque S A novel lysosome-associated membrane glycoprotein, DC-LAMP, induced upon DC maturation, is transiently expressed in MHC class II compartment Immunity 1998 9 325 336 10.1016/S1074-7613(00)80615-9 9768752

[B31] Defays A David A de Gassart A De Angelis Rigotti F Wenger T Camossetto V Brousset P Petrella T Dalod M Gatti E Pierre P BAD-LAMP is a novel biomarker of nonactivated human plasmacytoid dendritic cells Blood 2011 118 609 617 10.1182/blood-2010-11-319699 21642595

[B32] Rabinowitz SS Gordon S Macrosialin, a macrophage-restricted membrane sialoprotein differentially glycosylated in response to inflammatory stimuli J Exp Med 1991 174 827 836 10.1084/jem.174.4.827 1919437 PMC2118958

[B33] Salaun B de Saint-Vis B Clair-Moninot V Pin J-J Barthélemy-Dubois C Kissenpfennig A Peronne C Bates E Mattei M-G Lebecque S Cloning and characterization of the mouse homologue of the human dendritic cell maturation marker CD208/DC-LAMP Eur JImmunol 2003 33 2619 2629 10.1002/eji.200324175 12938238

[B34] Salaun B de Saint-Vis B Pacheco N Pacheco Y Riesler A Isaac S Leroux C Clair-Moninot V Pin JJ Griffith J Treilleux I Goddard S Davoust J Kleijmeer M Lebecque S CD208/dendritic cell-lysosomal associated membrane protein is a marker of normal and transformed type II pneumocytes Am J Pathol 2004 164 861 871 10.1016/S0002-9440(10)63174-4 14982840 PMC1613301

[B35] Debbabi H Ghosh S Kamath AB Alt J Demello DE Dunsmore S Behar SM Primary type II alveolar epithelial cells present microbial antigens to antigen-specific CD4^+ ^T cells Am J Physiol Lung Cell Mol Physiol 2005 289 L274 279 10.1152/ajplung.00004.2005 15833765

[B36] Gereke M Jung S Buer J Bruder D Alveolar type II epithelial cells present antigen to CD4(+) T cells and induce Foxp3(+) regulatory T cells Am J Respir Crit Care Med 2009 179 344 355 10.1164/rccm.200804-592OC 19096007

[B37] Wu Z Hu T Butter C Kaiser P Cloning and characterization of the chicken orthologue of dendritic cell-lysosomal associated membrane protein (DC-LAMP) Dev Comp Immunol 2010 34 183 188 10.1016/j.dci.2009.09.007 19782701

[B38] Wilke S Groebe L Maffenbeier V Jäger V Gossen M Josewski J Duda A Polle L Owens RJ Wirth D Heinz DW van den Heuvel J Büssow K Streamlining homogeneous glycoprotein production for biophysical and structural applications by targeted cell line development Plos One 2011 6 e27829 10.1371/journal.pone.0027829 22174749 PMC3235087

[B39] Wilke S Krausze J Gossen M Groebe L Jäger V Gherardi E van den Heuvel J Büssow K Glycoprotein production for structure analysis with stable, glycosylation mutant CHO cell lines established by fluorescence-activated cell sorting Protein Sci 2010 19 1264 1271 10.1002/pro.390 20512979 PMC2895251

[B40] Krissinel E Henrick K Inference of macromolecular assemblies from crystalline state J Mol Biol 2007 372 774 797 10.1016/j.jmb.2007.05.022 17681537

[B41] Murzin AG Brenner SE Hubbard T Chothia C SCOP: a structural classification of proteins database for the investigation of sequences and structures J Mol Biol 1995 247 536 540 7723011 10.1006/jmbi.1995.0159

[B42] Chan AW Hutchinson EG Harris D Thornton JM Identification, classification, and analysis of beta-bulges in proteins Protein Sci 1993 2 1574 1590 10.1002/pro.5560021004 8251933 PMC2142268

[B43] Tremblay DM Tegoni M Spinelli S Campanacci V Blangy S Huyghe C Desmyter A Labrie S Moineau S Cambillau C Receptor-binding protein of Lactococcus lactis phages: identification and characterization of the saccharide receptor-binding site J Bacteriol 2006 188 2400 2410 10.1128/JB.188.7.2400-2410.2006 16547026 PMC1428394

[B44] McMahon SA Miller JL Lawton JA Kerkow DE Hodes A Marti-Renom MA Doulatov S Narayanan E Sali A Miller JF Ghosh P The C-type lectin fold as an evolutionary solution for massive sequence variation Nat Struct Mol Biol 2005 12 886 892 10.1038/nsmb992 16170324

[B45] Jentoft N Why are proteins O-glycosylated? Trends Biochem Sci 1990 15 291 294 10.1016/0968-0004(90)90014-3 2204153

[B46] Eswar N Webb B Marti-Renom MA Madhusudhan MS Eramian D Shen MY Pieper U Sali A Comparative protein structure modeling using MODELLER Curr Protoc Bioinformatics 2006 Chapter 5 Unit 5 6 10.1002/0471250953.bi0506s15 PMC4186674 18428767

[B47] Bohne-Lang A von der Lieth CW GlyProt: in silico glycosylation of proteins Nucleic Acids Res 2005 33 W214 219 10.1093/nar/gki385 15980456 PMC1160146

[B48] GLYCAM Web http://www.glycam.com

[B49] Javadpour MM Eilers M Groesbeek M Smith SO Helix packing in polytopic membrane proteins: role of glycine in transmembrane helix association Biophys J 1999 77 1609 1618 10.1016/S0006-3495(99)77009-8 10465772 PMC1300449

[B50] Demirel Ö Jan I Wolters D Blanz J Saftig P Tampé R Abele R The lysosomal polypeptide transporter TAPL is stabilized by the interaction with LAMP-1 and LAMP-2 J Cell Sci 2012 in press 10.1242/jcs.087346 22641697

[B51] Schuske K Palfreyman MT Watanabe S Jorgensen EM UNC-46 is required for trafficking of the vesicular GABA transporter Nat Neurosci 2007 10 846 853 10.1038/nn1920 17558401

[B52] Griffiths G Back R Marsh M A quantitative analysis of the endocytic pathway in baby hamster kidney cells J Cell Biol 1989 109 2703 2720 10.1083/jcb.109.6.2703 2592402 PMC2115901

[B53] Griffiths G Fuller SD Back R Hollinshead M Pfeiffer S Simons K The dynamic nature of the Golgi complex J Cell Biol 1989 108 277 297 10.1083/jcb.108.2.277 2537312 PMC2115421

[B54] Schröder BA Wrocklage C Hasilik A Saftig P The proteome of lysosomes Proteomics 2010 10 4053 4076 10.1002/pmic.201000196 20957757

[B55] Reitsma S Slaaf D Vink H van Zandvoort M oude Egbrink M The endothelial glycocalyx: composition, functions, and visualization Pflügers Arch 2007 454 345 359 10.1007/s00424-007-0212-8 PMC1915585 17256154

[B56] Waterhouse AM Procter JB Martin DMA Clamp M Barton GJ Jalview Version 2--a multiple sequence alignment editor and analysis workbench Bioinformatics 2009 25 1189 1191 10.1093/bioinformatics/btp033 19151095 PMC2672624

[B57] Katoh K Asimenos G Toh H Multiple alignment of DNA sequences with MAFFT Methods Mol Biol 2009 537 39 64 10.1007/978-1-59745-251-9_3 19378139

[B58] Guex N Peitsch MC SWISS-MODEL and the Swiss-Pdb Viewer: An environment for comparative protein modeling Electrophoresis 1997 18 2714 2723 10.1002/elps.1150181505 9504803

[B59] Carlsson SR Lycksell PO Fukuda M Assignment of O-glycan attachment sites to the hinge-like regions of human lysosomal membrane glycoproteins lamp-1 and lamp-2 Arch Biochem Biophys 1993 304 65 73 10.1006/abbi.1993.1322 8323299

[B60] Case DA Darden TA Cheatham TE III Simmerling CL Wang J Duke RE Luo R Walker RC Zhang W Merz KM Roberts B Wang B Hayik S Roitberg A Seabra G Kolossvary I Wong FK Paesani F Vanicek J Liu J Wu X Brozell SR Steinbrecher T Gohlke H Cai Q Ye X Wang J Hsieh M-J Cui G Roe DR AMBER11 2010 San Francisco: University of California

[B61] Kirschner KN Yongye AB Tschampel SM Gonzalez-Outeirino J Daniels CR Foley BL Woods RJ GLYCAM06: a generalizable biomolecular force field. Carbohydrates J Comput Chem 2008 29 622 655 10.1002/jcc.20820 17849372 PMC4423547

[B62] Kabsch W XDS Acta Crystallogr D 2010 66 125 132 10.1107/S0907444909047337 20124692 PMC2815665

[B63] Matthews BW Solvent content of protein crystals J Mol Biol 1968 33 491 497 10.1016/0022-2836(68)90205-2 5700707

[B64] Vagin A Teplyakov A MOLREP: An automated programm for molecular replacement J Appl Crystallogr 1997 30 1022 1025 10.1107/S0021889897006766

[B65] Adams PD Afonine PV Bunkoczi G Chen VB Davis IW Echols N Headd JJ Hung LW Kapral GJ Grosse-Kunstleve RW McCoy AJ Moriarty NW Oeffner R Read RJ Richardson DC Richardson JS Terwilliger TC Zwart PH PHENIX: a comprehensive Python-based system for macromolecular structure solution Acta Crystallogr D 2010 66 213 221 20124702 10.1107/S0907444909052925 PMC2815670

[B66] McCoy AJ Grosse-Kunstleve RW Adams PD Winn MD Storoni LC Read RJ Phaser crystallographic software J Appl Crystallogr 2007 40 658 674 10.1107/S0021889807021206 19461840 PMC2483472

[B67] Emsley P Cowtan K Coot: model-building tools for molecular graphics Acta Crystallogr D 2004 60 2126 2132 10.1107/S0907444904019158 15572765

[B68] Pettersen EF Goddard TD Huang CC Couch GS Greenblatt DM Meng EC Ferrin TE UCSF Chimera--a visualization system for exploratory research and analysis J Comput Chem 2004 25 1605 1612 10.1002/jcc.20084 15264254

[B69] Kabsch W Sander C Dictionary of protein secondary structure: pattern recognition of hydrogen-bonded and geometrical features Biopolymers 1983 22 2577 2637 10.1002/bip.360221211 6667333

[B70] Ho BK Gruswitz F HOLLOW: generating accurate representations of channel and interior surfaces in molecular structures BMC Struct Biol 2008 8 49 10.1186/1472-6807-8-49 19014592 PMC2603037

